# Pre-clinical evaluation of a quadrivalent HCV VLP vaccine in pigs following microneedle delivery

**DOI:** 10.1038/s41598-019-45461-z

**Published:** 2019-06-25

**Authors:** D. Christiansen, L. Earnest-Silveira, B. Grubor-Bauk, D. K. Wijesundara, I. Boo, P. A. Ramsland, E. Vincan, H. E. Drummer, E. J. Gowans, J. Torresi

**Affiliations:** 1 Department of Microbiology and Immunology, The Peter Doherty Institute for Infection and Immunity, University of Melbourne, Parkville, Victoria, Australia; 2 Department of Surgery, The University of Adelaide and The Basil Hetzel Institute for Translational Health Research, Adelaide, South Australia Australia; 3 Burnet Institute, Melbourne, Victoria, Australia; 4 School of Science, College of Science, Engineering and Health, RMIT University, Melbourne, Victoria, Australia; 5 Department of Surgery Austin Health, University of Melbourne, Heidelberg, Victoria, Australia; 6 Department of Immunology, Central Clinical School, Monash University, Melbourne, Victoria, Australia; 7 The Peter Doherty Institute for Infection and Immunity, University of Melbourne, Parkville, Victoria, Australia; 8 Department of Microbiology, Monash University, Clayton, Australia

**Keywords:** Hepatitis C, Hepatitis C virus, Preclinical research

## Abstract

The introduction of directly acting antiviral agents (DAAs) has produced significant improvements in the ability to cure chronic hepatitis C infection. However, with over 2% of the world’s population infected with HCV, complications arising from the development of cirrhosis of the liver, chronic hepatitis C infection remains the leading indication for liver transplantation. Several modelling studies have indicated that DAAs alone will not be sufficient to eliminate HCV, but if combined with an effective vaccine this regimen would provide a significant advance towards achieving this critical World Health Organisation goal. We have previously generated a genotype 1a, 1b, 2a, 3a HCV virus like particle (VLP) quadrivalent vaccine. The HCV VLPs contain the core and envelope proteins (E1 and E2) of HCV and the vaccine has been shown to produce broad humoral and T cell immune responses following vaccination of mice. In this report we further advanced this work by investigating vaccine responses in a large animal model. We demonstrate that intradermal microneedle vaccination of pigs with our quadrivalent HCV VLP based vaccine produces long-lived multi-genotype specific and neutralizing antibody (NAb) responses together with strong T cell and granzyme B responses and normal Th1 and Th2 cytokine responses. These responses were achieved without the addition of adjuvant. Our study demonstrates that our vaccine is able to produce broad immune responses in a large animal that, next to primates, is the closest animal model to humans. Our results are important as they show that the vaccine can produce robust immune responses in a large animal model before progressing the vaccine to human trials.

## Introduction

Hepatitis C Virus (HCV) infects 2% of the world’s population and complications arising from chronic HCV remain the most common indication for liver transplantation. The advent of directly acting antiviral agents (DAAs) for chronic HCV has resulted in high cure rates for patients infected with all genotypes of HCV. However, re-infection remains an important problem, especially in individuals at high-risk for HCV infection^1^. In 2015 there were an estimated 1.75 million new HCV infections and 71 million living with HCV worldwide^2^ and transmission of HCV in several countries, like the United States, continues to rise^3–6^. Between 2014 and 2015 the number of reported cases of HCV in the US rose by 11%^6^. In Europe a similar pattern has emerged where the number of reported cases increased by 26% between 2006 and 2015^7^. In Australia the overall notification rate for HCV has remained unchanged in the past 5 years but the annual incidence of hepatitis C among people who inject drugs (PWID) tripled between 2014 and 2015^8^. In 2016 the number of cases of chronic hepatitis C (CHC) increased by 12% increase compared to 2015^9^. The overriding message from these data is that even with broader access to DAAs, a combination of approaches including multifaceted preventative risk-reduction strategies and ultimately an effective vaccine together with DAAs will be required to achieve the elimination of HCV^3^.

Although past resolved infection of HCV does not necessarily protect against reinfection, we know that clearance of HCV after reinfection is twice as likely compared to individuals who become infected for the first time. The rapid induction of cross neutralizing antibody (NAb) is also associated with viral clearance in these individuals^1,10,11^. These studies show that infected individuals develop protective immunity and so it should be possible to develop a vaccine that is able to prevent persistent infection.

Several HCV vaccine candidates have been studied to date. These have included recombinant proteins^12,13^, recombinant adenoviral and modified vaccinia Ankara (MVA), DNA and VLP vaccines in various prime boost approaches^14–19^ all with varying levels of success in producing potentially protective immune responses. To overcome the diversity of HCV an effective vaccine must be capable of generating both broad and cross-reactive NAb plus cell mediated immune (CMI) responses across multiple HCV genotypes circulating in nature^20,21^.

Although development of NAb is associated with protection against HCV, the importance of CD4^+^ and CD8^+^ T cells in both the clearance of and protection against HCV cannot be understated^22–24^. The core protein of HCV is one of the most important targets of protective CMI responses against HCV and its inclusion in any vaccine would therefore be highly desirable^22,24^.

A number of HCV VLP based vaccines producing HCV specific humoral and CMI responses have been described^13,18,25–31^. These vaccines have been shown to produce cross reactive NAb and T cell responses in various animal modes including mice and macaques and partial protection in chimpanzees^32,33^.

We have produced an HCV virus like particle (VLP) vaccine containing the HCV core and the E1 and E2 envelope glycoproteins and showed this vaccine induced NAb and T cell responses from a single vaccine construct^18,27,34,35^. We have also produced a quadrivalent HCV VLP vaccine containing particles of genotypes 1a, 1b, 2a and 3a^34^ and have shown that our vaccine produces strong antibody and T cell responses in mice^35,36^. Here we advance this work further by demonstrating that our quadrivalent vaccine produces robust immune responses in a pre-clinical large animal model that is of more relevance to human vaccination. Intradermal vaccination of Landrace pigs with quadrivalent VLP using a microneedle injector generated durable HCV multi-genotypic neutralizing antibody and CMI responses.

## Results

### Durable HCV VLP specific antibody responses following intradermal vaccination

**Figure 1:**
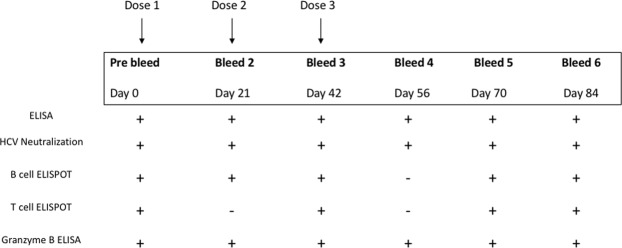
Overview of the HCV VLP quadrivalent vaccine immunisation, bleeding and testing schedule. Two groups of 3 pigs were immunized with 3 doses 21 days apart of quadrivalent vaccine by intradermal (Group 1) or intramuscular (Group 2) vaccination. Each dose consisted of a total of 300 µg of HCV VLP, containing 75 µg of each genotype. The assays performed at each sampling point are shown.

**Figure 2:**
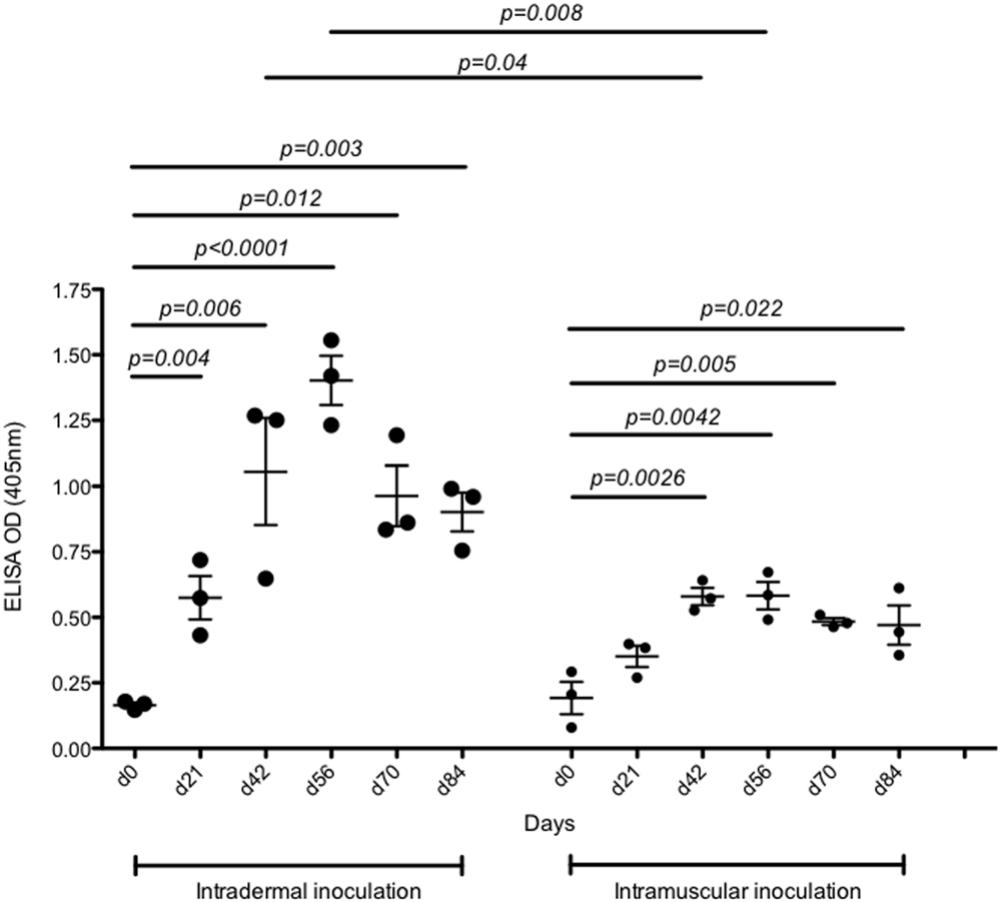
Antibody responses elicited by immunization with quadrivalent vaccine. White Landrace pigs (n = 3/group) were vaccinated with quadrivalent vaccine by intradermal microneedle injection or intramuscular injection. Antibody levels were determined by ELISA using heat-inactivated sera prepared from blood taken from all pigs at each of the indicated time points. Serum was diluted 1 in 500 in BSA_5_PBST and 50 µl of the diluted serum was added to VLP coated wells. The mean value and standard deviation is shown, this was calculated from the 3 pigs in each treatment group and is shown for each time point. P values in this, and other subsequent experiments were calculated using one-way ANOVA.

We have previously shown that both genotype 1a^25,34,35^ and 3a^18^ HCV VLP vaccines produced strong antibody responses in mice. To broaden the immunogenicity of the vaccine we subsequently produced a quadrivalent genotype 1a, 1b, 2a and 3a HCV VLP vaccine^27^ and demonstrated genotype specific immune responses in mice^36^. We have now advanced this work by testing the immunogenicity of our quadrivalent vaccine in a pre-clinical large animal model. Pigs were immunised 3 times, 21 days apart by intradermal or intramuscular injection and followed out to day 84-post inoculation (Fig. 1). These two routes of inoculation were selected as they represent two common routes of vaccine administration in humans. Sera collected at each time point were tested by ELISA to determine antibody responses (Fig. 2). Pigs in both groups developed an early antibody response at day 21. The responses peaked in both groups at day 56 and persisted to day 84, the last collection time point. Importantly, antibody responses were significantly higher at all time points tested in pigs vaccinated by the intradermal route when compared to intramuscular (Fig. 2).

**Figure 3:**
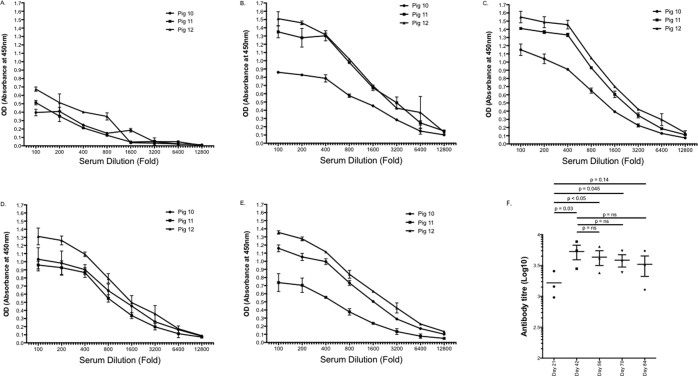
Time course of VLP specific antibody responses following quadrivalent vaccination via intradermal microneedle injection. Anti-VLP antibody levels at days 21 (**A**), 42 (**B**), 56 (**C**), 70 (**D**) and 84 (**E**) were determined by ELISA using serially diluted heat-inactivated sera from each of the individual pigs. The mean value and standard deviation is shown for triplicate samples for each dilution. Geometric mean antibody titres peaked at day 42 and were maintained through to day 84 (**F**).

As the intradermal group had superior antibody responses at all time points for the duration of the experiment, antibody responses were further characterised only in this group of pigs. Antibody responses were analysed for each collection time point for each of the individual pigs by ELISA (Fig. 3A–E). Lower absorbance values were observed from each of the individual pigs at d21 (Fig. 3A). The geometric mean titre (GMT) at day 21 was 3.18 log_10_ (95%CI 2.65 to 3.72). After d21 and the third dose of vaccine antibody titres increased significantly (Fig. 3B–E), reaching a peak for all pigs between d42 (GMT 3.74 log_10_ [95%CI 3.27 to 4.34] p = 0.03) and d56 (GMT 3.58 log_10_ [95%CI 3.12 to 4.11], p < 0.05) (Fig. 3B,C,F). Thereafter titres remained high at day 70 Fig. 3D (GMT 3.56 log_10_ 95%CI 3.14 to 4.04, p = 0.045) and persisted through to the completion of the experiment at day 84 (Fig. 3E) (GMT (3.43 log_10,_ 95%CI 2.69 to 4.36, p = 0.14)(Fig. 3F). Throughout the duration of the experiment antibody responses were typically highest for pig 12 compared to pigs 10 and 11.

**Figure 4:**
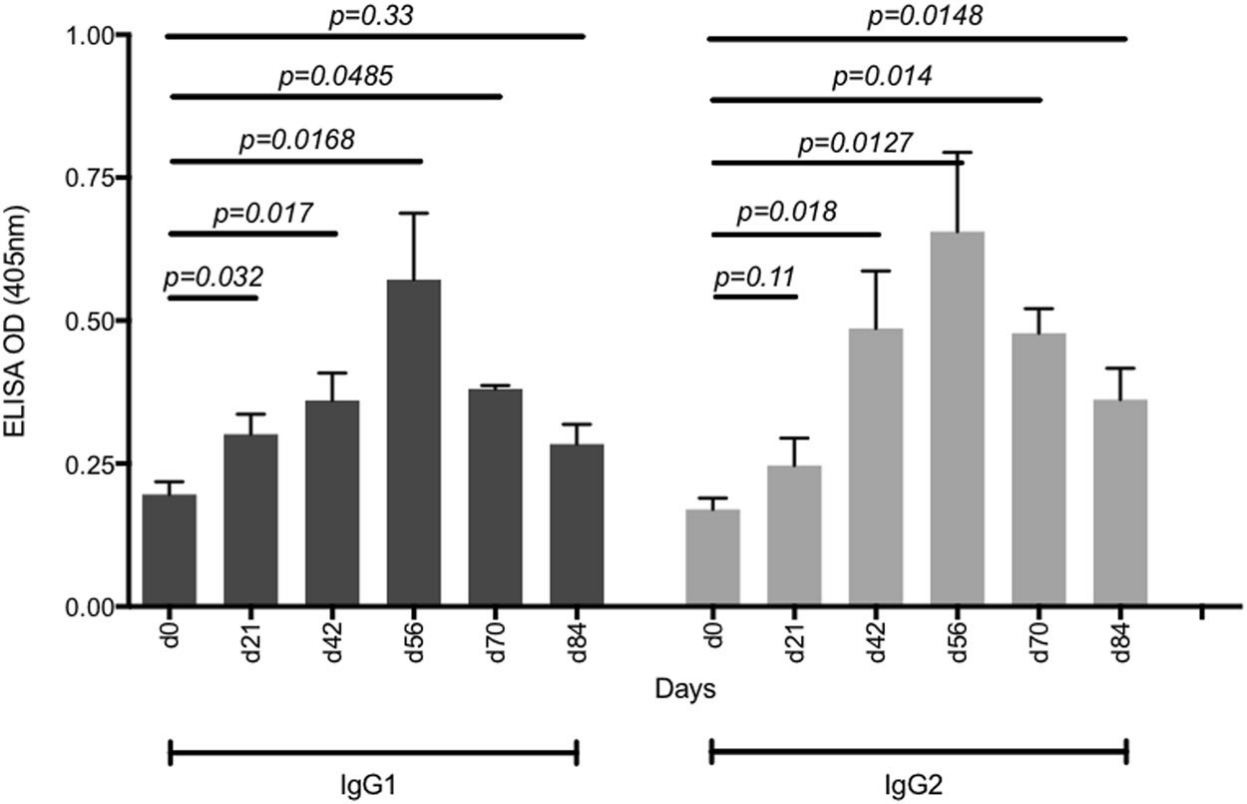
IgG1 and IgG2 responses generated after immunization with quadrivalent vaccine via intradermal microneedle injection. IgG1 and IgG2 antibody levels were determined by ELISA using heat-inactivated sera prepared from blood from all pigs at each time point. Serum was diluted 1 in 500 in BSA_5_PBST and 50 µl of the diluted serum was added to VLP coated wells. The mean value and standard deviation is shown, this was calculated from the 3 pigs in the group and is shown for each bleed.

We next determined the ability of the vaccine to induce the production of IgG1 and 2 subclasses as both have been shown to play important roles in protective humoral immune responses against viral infections^37–41^. We focused the analysis on the group inoculated by the intradermal route as this group produced the highest antibody titres. Both IgG1 and IgG2 were detected as early as day 21 after the first dose of the quadrivalent vaccine and they continued to significantly increase with each booster dose, peaking by day 56 and persisting to day 84 (Fig. 4).

### HCV genotype specific antibody responses following intradermal vaccination

We recently showed that our quadrivalent vaccine produced HCV genotype-specific antibody responses in mice^36^. Therefore, it was important to demonstrate that the same responses were possible in a pre-clinical large animal model. Furthermore, we also wanted to determine the long-term durability of the genotype specific response.

**Figure 5:**
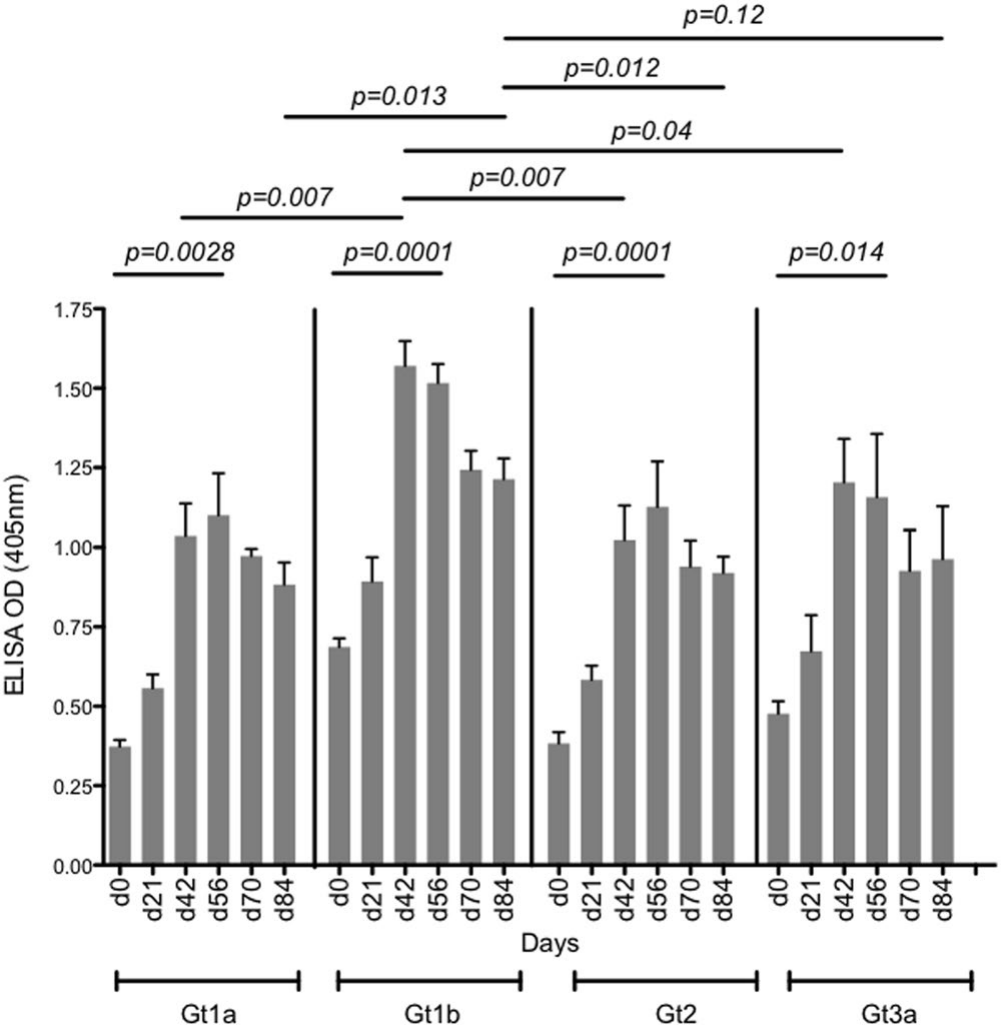
Genotype-specific antibody responses elicited by immunization with quadrivalent vaccine. Serum was diluted 1 in 500 in BSA_5_PBST and 50 µl of the diluted serum was added to genotype specific HCV VLP (Gt1a, Gt1b, Gt2a or Gt3a) coated wells. The mean value and standard deviation is shown, this was calculated from the 3 pigs at each time point.

Background antibody levels against each of the HCV genotypes were detected in pre-bleed sera on day 0 (Fig. 5, d0), however genotype specific antibody levels significantly increased following immunisation and were detected after a single vaccine dose (d21)(Fig. 5). The antibody responses peaked at days 42 (after dose 3) to 56 and persisted to day 84, remaining high for all four genotypes. Interestingly, responses at day 42 were significantly stronger for genotype 1b compared to genotypes 1a, 2a and 3a. Furthermore, the durability of responses to day 84 were also significantly stronger for genotype 1b compared to genotypes 1a, 2a and genotype 3a, which were all similar (Fig. 5).

### Quadrivalent HCV VLP vaccine induces broad neutralizing antibody responses

**Figure 6:**
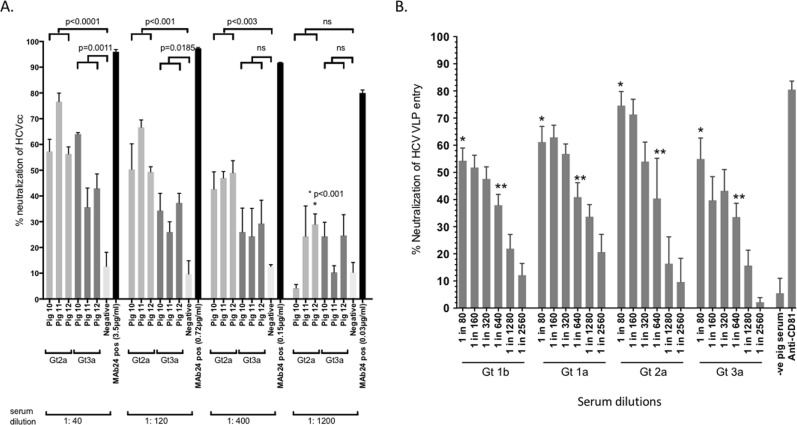
(**A**) Neutralizing antibody responses to quadrivalent VLP. Percentage HCV inhibition was determined by preincubating HCV infectious cell culture virus (HCVcc) genotype 2a or 3a with an equal amount of diluted immune serum from pigs inoculated by intradermal injection with quadrivalent VLP. Sera from the day 56 time point were used for all HCVcc neutralisation assays. Several serum dilutions (1:40, 1:140, 1:400 and 1:1200) were tested. The concentrations (μg/ml) of the MAb24 used for each dilution series are as indicated. The negative control was pig IgG purified from pooled serum collected from pigs 10–12 at day 0. Individual animals are presented for each group, with the mean value being represented by the horizontal bar. (**B**) Cross-neutralising antibody responses to quadrivalent VLP. Percent neutralization was determined by pre-incubating FITC labelled VLP (Gt1b, Gt1a, Gt2a or Gt3a) with increasing dilutions of day 56 serum (1:80 to 1:2560) from pigs 10–12. Anti-CD81 and non- immunized pooled pig IgG were used as positive and negative controls respectively. The mean value and standard deviation is shown, this was calculated from pigs 10–12 and is shown for each dilution. Immune serum compared to negative pooled pig serum; *p < 0.001; **p < 0.01.

Having shown that quadrivalent vaccine can produce strong antibody responses we then determined neutralizing antibody responses. Increasing dilutions of sera from the pigs immunized by the intradermal route were tested using cell culture derived Jc1 (genotype 2a) and genotype 3a HCVcc^42^. The sera from the vaccinated pigs inhibited Jc1 HCVcc entry by 56–77% (mean 64% +/− SD 11%) at a dilution of 1:40, 50–67% (mean 55% +/− SD 12%) at a dilution of 1:120 and 42–50% (mean 46% +/− SD 4%) at a 1:400 dilution (Fig. 6A). Further dilution of sera to 1:1200 resulted in little or no inhibition of HCVcc entry. Background levels of neutralization with pooled negative control pig serum were 10–16% (mean 12% +/− SD 8%), while the MAb24 positive control at all concentrations tested markedly inhibited HCVcc entry by 80–96% (mean 91% +/− SD 7%) (Fig. 6). Inhibition of entry was also observed with Gt3a, although the observed levels were somewhat lower across the three serum dilutions. Sera from the vaccinated pigs inhibited genotype 3a HCVcc entry by 36–64% (mean 48% +/− SD 15%) at a dilution of 1:40 but was less effective at higher dilutions, inhibiting entry by only 26–37% (mean 33% +/− SD 6%) at a dilution of 1:120 and 13–29% (mean 27% +/− SD 3%) at a 1:400 dilution (Fig. 6A).

### HCV VLP cross neutralizing responses

Having demonstrated strong neutralizing antibody responses to the available HCVcc Gt2a and Gt3a genotypes we next determined the cross-neutralization efficacy of our quadrivalent vaccine. HCV VLP from the four different genotypes were labeled with FITC and neutralization of HCV entry into Huh7 cells was tested using immune sera from vaccinated pigs and compared against pooled non-immune pig sera (Fig. 6B). At the lowest dilution (1:80), sera from pigs immunized with HCV VLPs neutralized genotype 1b VLPs by a mean of 55% (+/− SD 11%), compared with 61% (+/− SD 13%) for genotype 1a, 75% (+/− SD 12%) for genotype 2a and 55% (+/− SD 18%) for genotype 3a. Significant levels of neutralization against all four genotypes continued to be observed even at the higher serum dilutions (1:640 and 1:1280) (Fig. 6B). As expected the pooled non-immune pig serum only neutralized HCV VLPs by 6% (+/− SD 5%) while anti-CD81 antibody significantly neutralized HCV VLPs by 84% (+/− SD 6%).

**Figure 7:**
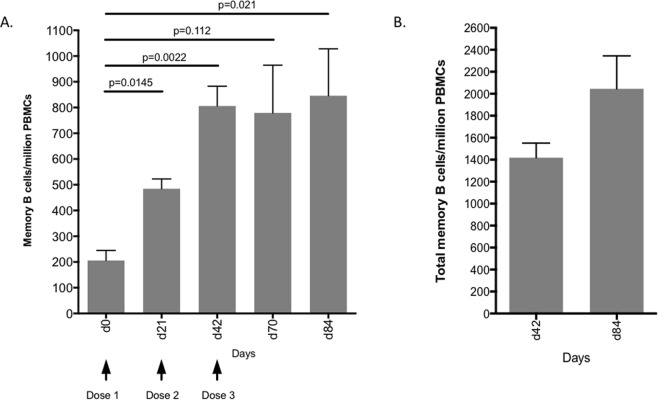
Memory B cell responses following immunization with quadrivalent vaccine. (**A**) PBMCs were harvested on each of the days as indicated from the 3 individual pigs (10–12) and frequencies of HCV specific memory B cells were determined by B cell ELISpot assay. The y-axis shows memory B cells number/million PBMCs. The mean value and standard deviation is shown. This was calculated from the 3 pigs and is shown for each time point. (**B**) The total number of IgG memory B cells on days 42 and 84 following R848 stimulation (a TLR9 agonist).

An important finding was that neutralizing antibody responses were strongest against genotype 2a in both the HCVcc and FITC-HCV VLP entry assays and responses were durable in all animals against all four serotypes out to day 84 (Supplementary figures 1–4).

### HCV VLP Specific B-cell responses

We next assessed B-cell responses following vaccination with quadrivalent VLP by determining the number of specific memory B cells using an ELISpot assay with HCV VLPs as the detecting antigen at time points with available PBMCs. A strong response was observed on day 21 after the first dose of vaccine (mean 485 +/−SD 66)/million cells) compared to pre-vaccination (d0) (mean 206 +/− SD 67)/million cells; p = 0.015) (Fig. 7A). The number of memory B cells increased significantly by day 42 (mean 806 +/− SD 133)/million cells) and were maintained through to days 70 (mean 779 +/− SD 320)/million cells) and 84 (6 weeks after the last vaccine dose) (mean 846 +/− SD 257)/million cells) (Fig. 7A). The total number of memory B cells at days 42 and 84 following R848 stimulation, a bovine/porcine TLR9 ligand^43^, was 1419 +/− SD 509/million cells and 2045 +/− SD 1163/million cells respectively (Fig. 7B). These results show that the quadrivalent VLP vaccine induces strong and durable HCV VLP-specific memory B cell responses. Plots for individual animals are included as a supplementary figure (Supplementary Fig. 5).

### Vaccination induces HCV VLP-specific T-cell, Granzyme B and cytokine responses

**Figure 8:**
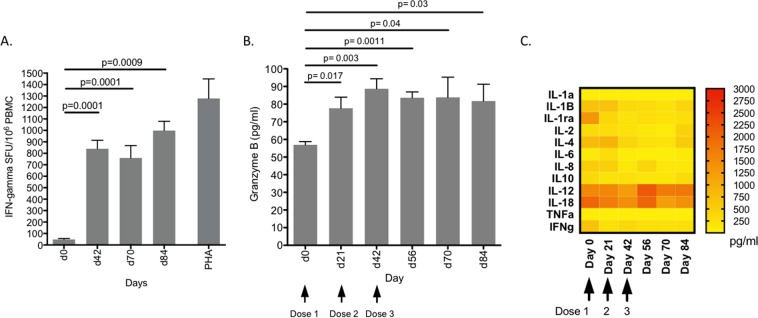
T cell response following immunization with quadrivalent vaccine (**A**) IFNγ T cell responses. Frequencies of IFNγ secreting cells were determined by ELISpot assay. The y-axis shows spots/million PBMCs. The mean value and standard deviation is shown, this was calculated from the 3 pigs at each bleed. (**B**) Granzyme B responses. The concentration of granzyme B (pg/ml) present in pig sera at the indicated time points was determined with reference to a standard curve. The mean value and standard deviation is shown from the 3 pigs at each of the time points. (**C**) Cytokine profiles. Serum cytokine profiles were analysed at each time point using a Porcine Cytokine 13-Plex Discovery Assay. Yellow to red colour gradation represents increasing concentration of each cytokine (pg/ml).

Aside from the induction of broad neutralizing multi-genotypic antibody responses, an effective HCV vaccine needs to induce strong T cell responses. Therefore, we assessed the ability and durability of the quadrivalent VLP vaccine in induction of VLP-specific T cell responses in a large animal model. Unfortunately, the initial d21 sample time point could not be tested due to limited sample availability. The mean number of VLP-specific T cells secreting IFNγ by day 42 was 840 +/− SD 181 SFU (Fig. 8A). Although a modest decrease was observed by day 70 (mean 759 SFU +/− SD 266, p = 0.0028) high VLP-specific T cell responses were maintained to day 84 post-vaccination (mean 998 SFU +/− SD 141/p = 0.0028) (Fig. 8A). Phytohemaglutinin (PHA) was used as positive control, producing a strong IFNγ T cell response (mean 1279 SFU +/− SD 295).

To determine the magnitude and durability of the VLP-specific cytotoxic T cell responses *in vivo* we used a porcine granzyme B ELISA quantifying the amount of granzyme B present in the serum samples obtained from vaccinated pigs^44^. The mean concentration of granzyme B, calculated after subtracting the prebleed value, was 21 pg/ml (+/−SD 3.2) at day 21 post vaccination (Fig. 8B). This increased to a maximum of 32 pg/ml (+/−SD 11) by day 42 and remained at 27 pg/ml (+/−SD 5.6) from d56 until the completion of the experiment at d84 (Fig. 8B). These results showed that the quadrivalent vaccine was able to produce durable cytotoxic T cell responses *in vivo*.

We also examined the cytokine profile in response to our vaccine using serum cytokine arrays (Fig. 8C). Vaccination did not appear induce inflammatory cytokine responses with the levels of IL-1a, 1b, 1ra, IL-6, TNFα or IFNγ below detection limits in the time points at which blood was available. In contrast, IL-12 levels were elevated from day 56 (p = 0.26) and IL-18 was transiently, but not significantly, elevated after the third dose of vaccine. These are interesting observations as IL-12 is important in initiating macrophage, NK and T cell responses that are important as a defence against viral infections. IL-18, together with IL12, is important in driving Th1 responses against viral pathogens. The vaccine did not induce an IL-10 response, an inhibitor of IL-12 and T cell responses to viruses. IL-4, an important cytokine for Th2 responses and antibody production was also upregulated soon after vaccination (Fig. 8C).

## Discussion

The elimination of chronic viral hepatitis has become an important goal of the WHO^2^. Although treatment with DAAs represents a major advance towards achieving this goal a number of modelling studies have indicated that DAAs alone will not be sufficient, however, if combined with an effective vaccine HCV elimination could potentially be achieved^45–49^.

To this end, we have shown that our quadrivalent HCV VLP vaccine produces strong humoral and cell-mediated immune responses in a pre-clinical large animal model. Pigs are recognised as the closest animal model to humans, next to primates; as the porcine immune system more closely resembles humans than mice^50^. In addition, functional orthologs for cytokines involved in Th1, Th2 and Th17 responses and the corresponding cells have also been described in pigs^51–53^. In contrast to mice, young pigs also provide a large animal model with body weights that can be approximated to those of humans, an important consideration when trying to determine suitable vaccine doses. Hence, they serve as an ideal preclinical model to test the translational potential of our vaccine.

Our results show that the quadrivalent VLP vaccine administered intradermally without an adjuvant results in significantly stronger antibody responses than when given by intramuscular injection. The vaccine also produced long-lived B cell and genotype specific antibody responses and NAb response against genotypes 1a, 1b, 2a and 3a HCV. The ability of the vaccine to produce these responses is analogous to hepatitis B vaccine, which can also produce long term protective antibody responses in the absence of adjuvant^54,55^. We know that the induction of NAb is associated in protection against HCV infection in chimpanzees, humanised uPa-SCID liver chimeric mice and humans^11,56^ and so our results in pigs is an important finding as it reaffirms that our vaccine is able to produce such a response in a large animal.

The importance of NAb to HCV has been borne out in several studies showing that polyclonal and monoclonal antibodies directed to conformational neutralizing epitopes protect human liver chimeric uPA/SCID mice against challenge with HCV^57^. The most potent neutralizing human monoclonal antibodies (HuMAbs) recognise critical epitopes in the E1E2 heterodimers presented as complex tertiary or quaternary conformational structures on the surface of HCV^58–65^.

These potent HuMAbs predominantly recognize epitopes in domains B and D of the E2 protein, while HuMAbs binding to domain C are only weakly neutralizing^58–60^. Other potent neutralizing HuMAbs have also been described that target discontinuous epitopes outside domains B, C and D^62,66^. It appears that the development of NAb may arise from public B cell clonotypes that recognize conserved epitopes and that these NAb possess a substantial breadth of neutralizing activity that may also include the early autologous transmitted/founder viruses^67^. Interestingly, broad NAb can also be detected up to 25 years after spontaneous clearance of HCV, suggesting that the development of long-lived memory responses is possible^68^.

However, neutralization of all strains of HCV cannot be achieved with a single NAb as shown by recent studies reporting that several HuMAbs targeting different E2 domains can act synergistically to enhance neutralization of diverse strains of HCV compared to any individual NAb^69,70^. Several studies have approached NAb vaccines by designing recombinant antigens containing molecular scaffolds to present a limited number of E2 epitopes in a vaccine construct^71^ or developing truncated E2 proteins with deletion of variable regions to better expose conserved neutralization epitopes^72,66^. Whilst these vaccines are able to induce NAb production the recent studies reporting synergistic activity of different NAbs would argue that vaccination with full-length E1E2 as glycoprotein heterodimers or as VLPs presenting conformational epitopes may be advantageous since this vaccine is more likely to induce the production of combinations potent synergistically acting NAb to substantially enhance the breadth of neutralization against diverse strains of HCV^69,70^. This is analogous to the mechanism of neutralization described for flaviviruses like dengue^73,74^ and has important implications for vaccine design as an effective HCV vaccine will need to deliver multiple conformational epitopes that will result in the production of NAb with broad binding specificities^70^. HCV VLPs have the ability to achieve this goal and are therefore an attractive vaccine candidate for HCV^20,21,25,26,32,33^.

We have previously shown that our quadrivalent vaccine produces strong T cell and granzyme B responses in mice^35,36,25,34^. In this study we have shown a similar response in pigs, together with a favorable cytokine profile. The importance of both CD4^+^ and CD8^+^ T cell responses in clearance of and protection against HCV has been borne out by numerous studies^17,22,24,75–78^. In humans, strong and broad HCV core specific T cell responses are important in spontaneous viral clearance^22–24^. In contrast to persistent infection, spontaneous resolution of HCV infection in humans has been temporally linked to the appearance of strong, long-lived, polyfunctional CD4^+^ and CD8^+^ T cell responses that are directed against multiple HCV antigens and particularly the core protein^79^. Furthermore, we and others have shown that the potency of cytolytic T cell responses are crucial for protection against HCV^19,77^. In chimpanzees, CD4^+^ and CD8^+^ T cell depletion results in HCV persistence after viral challenge^75^ and rechallenge with homologous virus after spontaneous clearance results in the rapid re-expansion of HCV-specific CD8^+^ T cells in both blood and the liver and clearance of viraemia^76^. Our quadrivalent vaccine will deliver antigens capable of inducing HCV specific T cell responses in a large animal.

The advantage of presenting multiple HCV antigens, as a quadrivalent HCV VLP vaccine does, is also highlighted by a number of studies. The co-administration of a mixture of recombinant genotype 1b HCV core, E1, E2 proteins has been shown to induce strong HCV core specific T cell responses in vaccinated mice and African green monkeys^12^. Furthermore, vaccinated mice were able to control viremia after challenge with vaccinia virus expressing HCV structural proteins (vvRE)^12^. Recombinant human adenovirus 6 (Ad6) and chimpanzee adenovirus 3 (ChAd3) vaccines containing HCV non-structural genes have also been shown to produce long-lived polyfunctional central and effector memory CD4^+^ and CD8^+^ T cell responses against multiple homologous and heterologous (genotype 1a and 3a) HCV proteins^16^. In humans, a heterologous prime-boost vaccination strategy with ChAd3 and MVA vectors encoding the non-structural proteins of HCV genotype 1b produced high levels of polyfunctional CD8^+^ and CD4^+^ HCV-specific T cells targeting multiple homologous and heterologous HCV antigens together with sustained memory and effector T cell populations^17^. The limitation with these vaccines, however, is that unlike HCV VLPs, they do not produce NAb responses, a critical part of protection against HCV. Finally, it has also been shown that T cell responses of individuals with chronic hepatitis C infection are likely to be genotype specific with limited cross reactivity^80,81^. This would argue that, as with NAb, an effective vaccine will need to contain T cell antigens of more than a single genotype.

Our study also demonstrated that the administration of the quadrivalent vaccine by the intradermal route using a microneedle device resulted in stronger antibody response than the intramuscular route. The inoculation of vaccines using microneedle devices or intradermal injection has been shown to produce strong immune responses^82–85^. The delivery of vaccines to the skin using intradermal injection and microneedle devices presents antigens directly to unique populations of resident cutaneous antigen presenting cells (APCs), ensures a more accurate, effective and reproducible delivery of vaccine to the dermis and has an antigen sparing benefit^85^. The delivery of hepatitis B vaccine by intradermal injection for example has been shown to result in higher seroconversion rates and antibody titres than intramuscular inoculation^86^. Also, intradermal inoculation of hepatitis B vaccine results in higher seroconversion rates and antibody titres than intramuscular inoculation in patients with chronic renal failure^87^ and chronic liver disease^88^. Microneedles also allow for the delivery of multiple antigens^89^. A more effective production of protective influenza antibody responses has been reported following intradermal microneedle inoculation of vaccine^85^. Immunization of mice with influenza H5 VLPs by microneedle device produces durable humoral and cellular immune responses, including IgG1 and IgG2a antibody isotypes, consistent with both Th1 and Th2 responses^90^. These responses also translated to better protection against challenge with H5N1 avian influenza compared to intramuscular vaccination^91^. Vaccination of pigs with the quadrivalent vaccine also resulted in both IgG1 and 2 antibody responses. Similarly, intradermal influenza vaccine has also been shown to produce strong seroprotective antibody responses in humans^92^. Microneedle devices have been developed for the effective delivery of live viral vaccines like measles^93^. These investigators reported the development of long-lasting measles NAb responses in macaques following intradermal inoculation with a microneedle. These findings are similar to the responses we observed in pigs given the quadrivalent vaccine by intradermal route with a microneedle.

In conclusion, a vaccine to prevent persistent infection with HCV is not only an achievable goal but one that would also make a significant addition to the armamentarium required to eliminate hepatitis C globally. Our quadrivalent HCV VLP vaccine holds promise as it is able to produce long-lived broad genotype specific and NAb responses in a large animal model together with strong T cell and granzyme B responses and a normal Th1 and Th2 cytokine response. Based on these findings and our previous report demonstrating that critical epitopes for neutralization are expressed on the particles, our quadrivalent HCV VLP vaccine fulfills the requirements expected of an effective HCV vaccine candidate.

## Methods

### Production of HCV VLPs

Construction of recombinant adeno-encoding HCV structural proteins core E1 and E2 and the large-scale production, purification and characterisation of the quadrivalent genotype 1a, 1b, 2a, and 3a HCV VLP vaccine used in this study has been reported previously^25,27,34^. MAb24 is a murine NAb that recognizes a linear epitope in the E2_661_ protein that lacks the HVR1, HVR2 and igVR regions^72^.

### Protocol for immunization of pigs

White Landrace pigs were obtained from the University of Adelaide Roseworthy Campus and were housed at the Large Animal Research Facility, South Australian Health and Medical Research Institute. Vaccination studies were performed in two groups of three 6-week old outbred White Landrace pigs comparing intradermal (ID) and intramuscular (IM) routes of inoculation (Fig. 1). To ensure reproducible intradermal delivery of quadrivalent VLP, the immunisations were performed using a microneedle device (FluGen Inc, Wisconsin, USA) which delivered the VLP in a volume of 250 μl of saline. For group 1, White Landrace pigs were vaccinated with the quadrivalent vaccine by intradermal microneedle injection while group 2 were vaccinated by intramuscular injection, both without adjuvant. Pigs in each group received three doses of 75 µgs per HCV VLP genotype (total of 300 µgs of HCV VLPs) vaccine at 21-day intervals in the upper inner front leg. The pigs were bled aseptically through the jugular vein and blood processed for serum and PBMCs at baseline (d0), before each booster (days 21 and 42) and again on days 56, 70 and 84.

The University of Adelaide and the South Australia Pathology Animal Ethics Committees approved all experimental protocols (Animal Ethics Committee approval number M-2012-177). All procedures adhered to the Australian code for the care and use of animals for scientific purposes 8th edition 2013, Australian Government, National Health and Medical Research Council.

### Preparation of porcine PBMCs

PBMC were isolated from peripheral blood using Lymphoprep (StemCell Technologies) as per manufacturer’s instructions. Briefly, blood was diluted with an equal amount of Dulbecco’s Phosphate-Buffered Saline with 2% Fetal Bovine Serum (PBS + 2% FBS) and layered on top of Lymphoprep, followed by centrifugation at 800 × g for 20 minutes at room temperature (15–25 °C) without the brake applied. The PBMC interface was carefully removed by pipetting and washed with PBS + 2% FBS by centrifugation at 300  ×  g for 10 min. Cell number and viability were determined using a Countess Automated Cell Counter (Invitrogen). PBMC were cryopreserved in liquid nitrogen in 90% FCS containing 10% dimethyl sulfoxide (DMSO; Sigma Aldrich) and stored until required for downstream analyses.

### Enzyme-linked immunosorbent assay

Anti-HCV VLP antibody titres were determined by ELISA as previously described^25,36^. In brief, for VLP-specific antibody responses, flat bottom 96-well polyvinyl plates were coated with purified quadrivalent VLPs (5 µg/ml) in carbonate coating buffer (100 mM Na_2_CO_3_, and NaHCO_3_, pH 9.6) overnight at 4 °C. The plates were then blocked with 100 µl of BSA (10 mg/ml) in PBS and incubated for 1 hour at 37 °C before washing four times with PBST (PBS containing 0.05%^v/v^ Tween-20). For titration of antibody response heat inactivated serum was serially diluted 0.5-log_10_ in BSA (5 mg/ml) in PBST and incubated for 1 hour at 37 °C. To determine the strength of antibody responses at different time points, pig serum was diluted 1 in 500 in BSA_5_PBST and 50 µl of the diluted serum was added to the wells of a VLP coated plate. Identical serum dilutions and conditions were also used for determination of genotype-specific antibody and IgG1 and 2 responses. Absorbance values were determined on a Labsystems Multiskan Multisoft plate reader at 450 nm. Titres of antibody are expressed as the reciprocal of the highest dilution of serum required to achieve an optical density of 0.2.^25,42^.

### HCV Neutralization assay

Neutralization assays against HCV infectious cell culture system (HCVcc) were performed by mixing HCVcc virus with an equal volume of serially diluted immune serum, using methods as previously described^36,94^. Sera from the day 56 time point were used for all HCVcc neutralization assays. Each experiment was performed in triplicate. The virus/serum mixture was incubated for 1 h at 37 °C before addition to Huh7.5 cells that were seeded 24 h earlier at 30,000 cells/well in 48 well plates for 4 h. Cells were washed at least 4 times and replenished with fresh DMF10NEAA and incubated for a further 48–72 h. Luciferase activity was measured in clarified lysates using Renilla luciferase substrate (Promega) and a FLUOstar Optima microplate reader fitted with luminescence optics (BMG Life Technologies, Germany). The neutralization titre was calculated from 6-point dilution curves as the reciprocal dilution of serum to reduce luciferase activity by 50% (ID50). The data shown is the mean from at least two independent experiments. MAb24, used as a positive control for inhibition of HCVcc entry, is a murine NAb that recognizes a linear epitope in the modified recombinant E2_661_ protein that lacks the HVR1, HVR2 and igVR regions. The MAb24 antibody is produced using a Miniperm bioreactor, with a final concentration of the antibody of 150 µg/ml. The antibody is diluted 1 in 8 (18 µg/ml) and then used in a series of 0.5 log_10_ dilutions^72^. Pig IgG purified from pooled non-immune serum was used as the negative control.

The inhibition of entry of HCV VLPs into Huh7 cells by immune pig sera was determined by flow cytometry (FACS Canto II, BD Bioscience) using FITC labelled HCV VLP and analysed using the WEASEL 2.0 software package (Walter and Eliza Hall Institute, Melbourne, Australia) as described previously^34^. Sera that demonstrated a decrease in specific cellular binding of 50% or more were considered neutralizing. As a positive control, inhibition of entry of FITC-HCV VLPs into Huh7 cells was also determined using anti-CD81 antibody (Abcam, USA).

### HCV specific B Cell responses

For the detection of specific antibody secreting cells by ELISpot, Pig PBMCs were stimulated with R848, a TLR9 agonist^43^ (Invivogen, USA) for five days, cultured on ELISPOT plates and developed using a standardized Porcine IgG ELISpot^BASIC^ (HRP) ELISpot kit (Mabtech Inc., Cincinnati, OH, USA). Briefly, polyvinylidene fluoride (PVDF) membrane-lined 96-well plates (Millipore, Ireland) were coated with 100 µl of PBS containing quadrivalent VLPs (4 µg/well or 40 µg/ml) as previously described^25,36,95^ or 15 µg/ml of anti-Pig IgG (monoclonal antibody MT421). The plates were incubated overnight at 4 °C, washed with PBS, and blocked using complete R10 medium for 30 minutes. Stimulated PBMCs were then added to the ELISPOT wells and cultured in the plates overnight at 37 °C with 5% CO_2_. After 24 hours, wells were washed and biotinylated anti-IgG (monoclonal antibody MT424) antibody was added for 2 hours at room temperature. This was followed by streptavidin-conjugated horse-radish peroxidase (both from Mabtech, USA) and incubating for 1 hour at room temperature and detected with TMB substrate. Individual spots were then scanned, counted and analysed on an AID EliSpot Reader (Strassberg, Germany).

### Analysis of IFN-γ-secreting Cells

ELISpot assays were carried out using the IFN-γ ELISpot^BASIC^ (HRP) kit (Mabtech, USA) with PVDF membrane-lined 96-well plates (Millipore, Ireland) as described previously^36^. Wells were precoated with anti-IFN-γ capture monoclonal antibody pIFNγ-I at 10 µg/ml. Following coating, plates were then blocked with RF 10 medium. PBMCs stimulated for 5 days with 20 µg total VLPs per well were harvested, washed and serial dilutions commencing at 1 × 10^5^ cells/ml added to the wells. Forty-eight hours later, plates were washed with PBS and biotinylated anti-IFN-γ capture antibody (P2C11-biotin) was added and incubated for 2 hours at room temperature in a humidified atmosphere. Plates were then washed and streptavidin conjugated horse-radish peroxidase (Mabtech, USA) was added and incubated for one hour. Spots representative of IFN-γ-producing cells were developed by the addition of TMB.

### Analysis of granzyme B-secreting cells

For the detection of Granzyme B-secreting cells, a Porcine Granzyme B (GZMB) ELISA Kit (MyBioSource, USA) was used according to the manufacturer’s instructions. ELISA strip wells, precoated with Porcine GZMB monoclonal antibody were used for test samples and standards. Porcine GZMB standards were set up to give final concentrations of 1000, 500, 250, 125, 62.5, 31.2,15.6 pg/ml. Porcine sera was added in triplicates to the wells, followed by biotinylated polyclonal porcine GZMB antibody (1:100) and detected with the Colour Reagent provided by the manufacturer. Absorbance values were determined on a Labsystems Multiskan Multisoft plate reader at 450 nm and a standard curve was used to quantify the GZMB (pg/ml).

### Cytokine arrays

Serum cytokine profiles following vaccination were analysed using a Porcine Cytokine 13-Plex Discovery Assay (Eve Technologies, Calgary, Canada).

### Statistical analysis

Statistical analysis was performed using the Prism 5.0 software (GraphPad)^36^. In all cases the mean ± standard deviation of the mean (SD) is shown unless otherwise stated. P values for statistical analysis were calculated using one-way ANOVA with Tukey’s multiple comparison test. Differences were considered statistically significant when p values were less than 0.05 (p < 0.05) with a 95% confidence level.

## Supplementary information

Supplementary information
