# Supplementary material for: Pre-clinical evaluation of a quadrivalent HCV VLP vaccine in pigs following microneedle delivery
Source: Sci Rep. 2019 Jun 25;9:9251. doi: 10.1038/s41598-019-45461-z (PMC6592879; doi:10.1038/s41598-019-45461-z)
Supplement: Supplementary file 1 — Supplementary information [file 41598_2019_45461_MOESM1_ESM.pdf]

Pre-clinical evaluation of a quadrivalent HCV VLP vaccine in pigs following microneedle delivery

D Christiansen[1], L Earnest-Silveira[1], B Grubor-Bauk[2], DK Wijesundara[2], I Boo[3], PA Ramsland[3,4,5,6], E Vincan[7], HE Drummer[3,8], EJ Gowans[2], J Torresi[1]

[1]Department of Microbiology and Immunology, The Peter Doherty Institute for Infection and Immunity, University of Melbourne, Parkville, Victoria, Australia

[2]Department of Surgery, The University of Adelaide and The Basil Hetzel Institute for Translational Health Research, Adelaide, South Australia, Australia

[3]Burnet Institute, Melbourne, Victoria, Australia

[4]School of Science, College of Science, Engineering and Health, RMIT University, Melbourne, Victoria, Australia

[5]Department of Surgery Austin Health, University of Melbourne, Heidelberg, Victoria, Australia

[6]Department of Immunology, Central Clinical School, Monash University, Melbourne, Victoria, Australia

[7]The Peter Doherty Institute for Infection and Immunity, University of Melbourne, Parkville, Victoria, Australia

[8]Department of Microbiology, Monash University, Clayton, Australia

Supplementary Figure 1

Cross-neutralization of HCV Gt1a VLP entry into Huh7 cells. Inhibition was determined following preincubation of FITC labelled Gt1a VLP with pig serum (diluted 1:80-1:2560) collected at each time point throughout the duration of the experiment. Light to dark green colour gradation represents percentage inhibition, with the darker shades showing higher neutralization. Numerical values represent the mean value calculated from triplicate samples at each time point.

Supplementary Figure 2

Cross-neutralization of HCV Gt1b VLP entry into Huh7 cells.

Supplementary Figure 3

Cross-neutralization of HCV Gt2a VLP entry into Huh7 cells.

Supplementary Figure 4

Cross-neutralization of HCV Gt3a VLP entry into Huh7 cells.

Supplementary Figure 5

B cell response from individual pigs following immunization with quadrivalent vaccine. ELISpot assay showing the number of memory B cells for individual pigs from the start (d0) through to the completion of the experiment (d84). The mean value (black circle) and standard deviation is shown, calculated from triplicate samples at each time point.

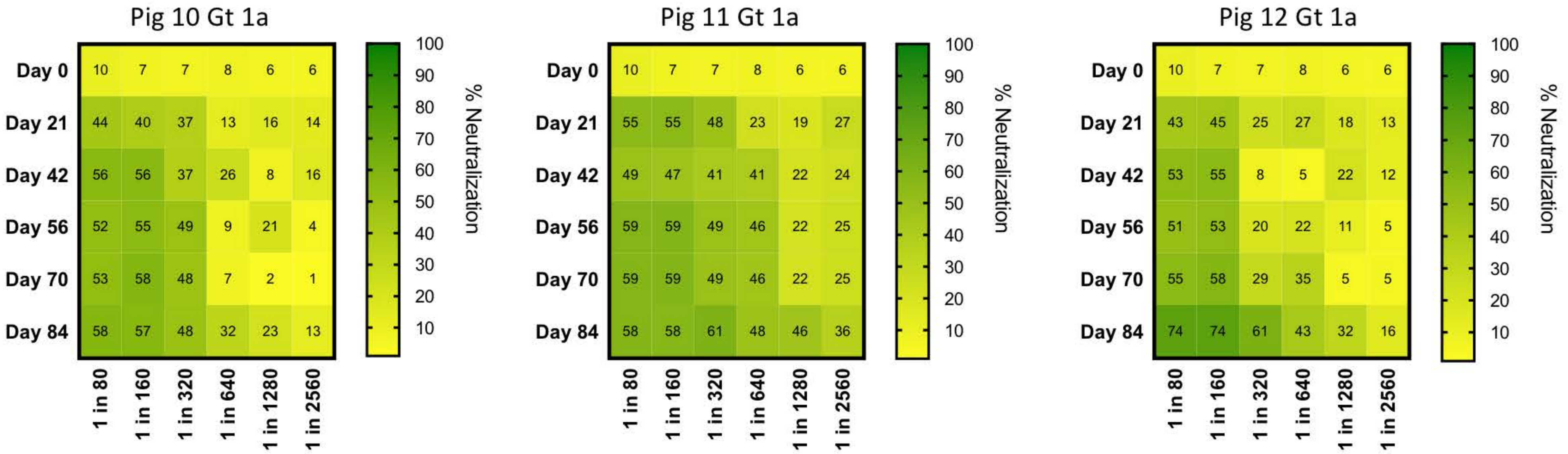

Pig 10 Gt 1a	1 in 80	1 in 160	1 in 320	1 in 640	1 in 1280	1 in 2560
Day 0	10	7	7	8	6	6
Day 21	44	40	37	13	16	14
Day 42	56	56	37	26	8	16
Day 56	52	55	49	9	21	4
Day 70	53	58	48	7	2	1
Day 84	58	57	48	32	23	13

Pig 11 Gt 1a	1 in 80	1 in 160	1 in 320	1 in 640	1 in 1280	1 in 2560
Day 0	10	7	7	8	6	6
Day 21	55	55	48	23	19	27
Day 42	49	47	41	41	22	24
Day 56	59	59	49	46	22	25
Day 70	59	59	49	46	22	25
Day 84	58	58	61	48	46	36

Pig 12 Gt 1a	1 in 80	1 in 160	1 in 320	1 in 640	1 in 1280	1 in 2560
Day 0	10	7	7	8	6	6
Day 21	43	45	25	27	18	13
Day 42	53	55	8	5	22	12
Day 56	51	53	20	22	11	5
Day 70	55	58	29	35	5	5
Day 84	74	74	61	43	32	16

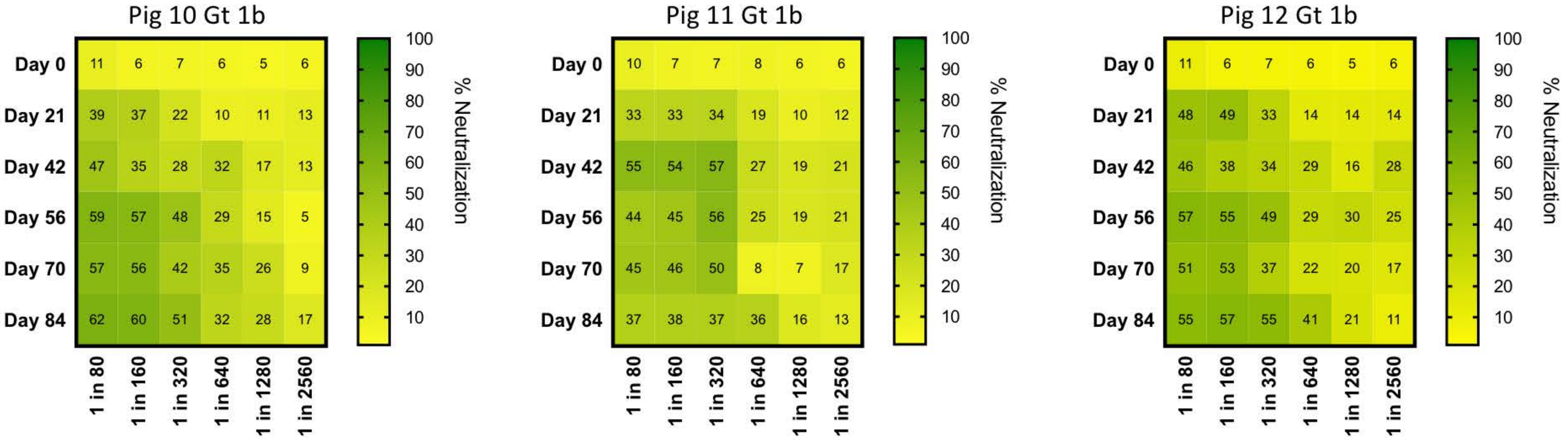
Pig 10 Gt 1b
Day 0 11 6 7 6 5 6
Day 21 39 37 22 10 11 13
Day 42 47 35 28 32 17 13
Day 56 59 57 48 29 15 5
Day 70 57 56 42 35 26 9
Day 84 62 60 51 32 28 17
1 in 80
1 in 160
1 in 320
1 in 640
1 in 1280
1 in 2560
100 90 80 70 60 50 40 30 20 10
% Neutralization
Pig 11 Gt 1b
Day 0 10 7 7 8 6 6
Day 21 33 33 34 19 10 12
Day 42 55 54 57 27 19 21
Day 56 44 45 56 25 19 21
Day 70 45 46 50 8 7 17
Day 84 37 38 37 36 16 13
1 in 80
1 in 160
1 in 320
1 in 640
1 in 1280
1 in 2560
100 90 80 70 60 50 40 30 20 10
% Neutralization
Pig 12 Gt 1b
Day 0 11 6 7 6 5 6
Day 21 48 49 33 14 14 14
Day 42 46 38 34 29 16 28
Day 56 57 55 49 29 30 25
Day 70 51 53 37 22 20 17
Day 84 55 57 55 41 21 11
1 in 80
1 in 160
1 in 320
1 in 640
1 in 1280
1 in 2560
100 90 80 70 60 50 40 30 20 10
% Neutralization

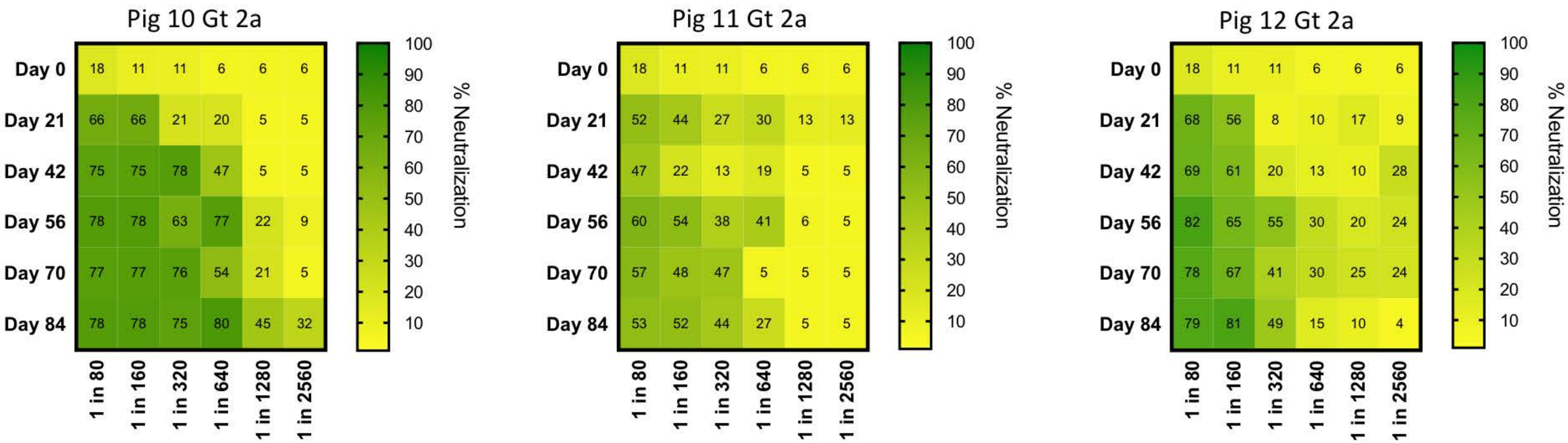
Pig 10 Gt 2a
Day 0
Day 21
Day 42
Day 56
Day 70
Day 84
18 11 11 6 6 6
66 66 21 20 5 5
75 75 78 47 5 5
78 78 63 77 22 9
77 77 76 54 21 5
78 78 75 80 45 32
1 in 80
1 in 160
1 in 320
1 in 640
1 in 1280
1 in 2560
100
90
80
70
60
50
40
30
20
10
% Neutralization
Pig 11 Gt 2a
Day 0
Day 21
Day 42
Day 56
Day 70
Day 84
18 11 11 6 6 6
52 44 27 30 13 13
47 22 13 19 5 5
60 54 38 41 6 5
57 48 47 5 5 5
53 52 44 27 5 5
1 in 80
1 in 160
1 in 320
1 in 640
1 in 1280
1 in 2560
100
90
80
70
60
50
40
30
20
10
% Neutralization
Pig 12 Gt 2a
Day 0
Day 21
Day 42
Day 56
Day 70
Day 84
18 11 11 6 6 6
68 56 8 10 17 9
69 61 20 13 10 28
82 65 55 30 20 24
78 67 41 30 25 24
79 81 49 15 10 4
1 in 80
1 in 160
1 in 320
1 in 640
1 in 1280
1 in 2560
100
90
80
70
60
50
40
30
20
10
% Neutralization

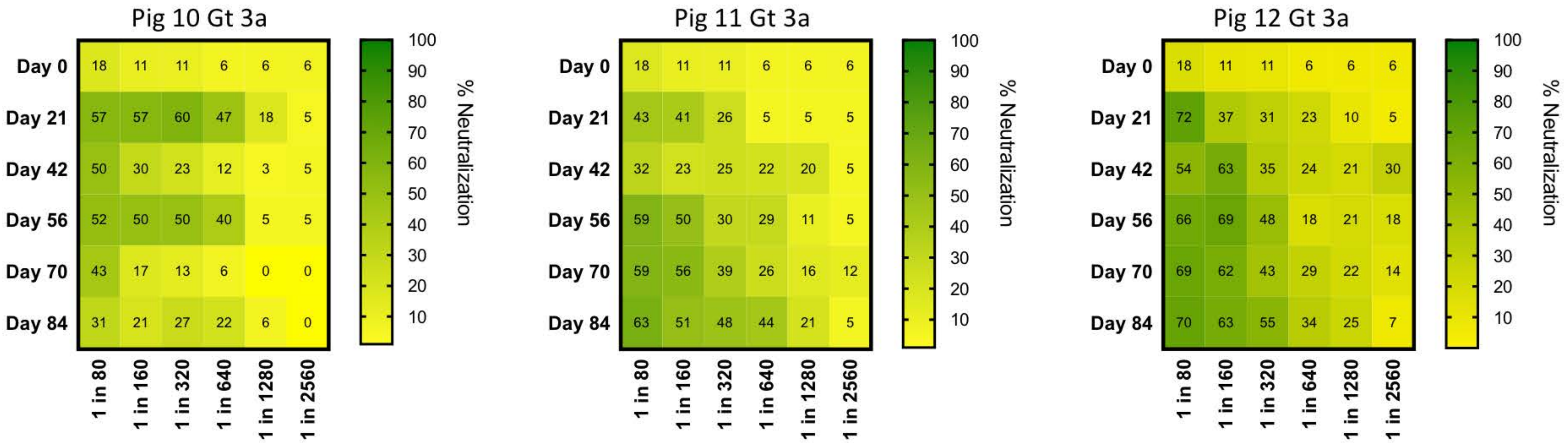
Pig 10 Gt 3a
Pig 11 Gt 3a
Pig 12 Gt 3a
Day 0
Day 21
Day 42
Day 56
Day 70
Day 84
1 in 80
1 in 160
1 in 320
1 in 640
1 in 1280
1 in 2560
% Neutralization
18 11 11 6 6 6
57 57 60 47 18 5
50 30 23 12 3 5
52 50 50 40 5 5
43 17 13 6 0 0
31 21 27 22 6 0
18 11 11 6 6 6
43 41 26 5 5 5
32 23 25 22 20 5
59 50 30 29 11 5
59 56 39 26 16 12
63 51 48 44 21 5
18 11 11 6 6 6
72 37 31 23 10 5
54 63 35 24 21 30
66 69 48 18 21 18
69 62 43 29 22 14
70 63 55 34 25 7
10 20 30 40 50 60 70 80 90 100

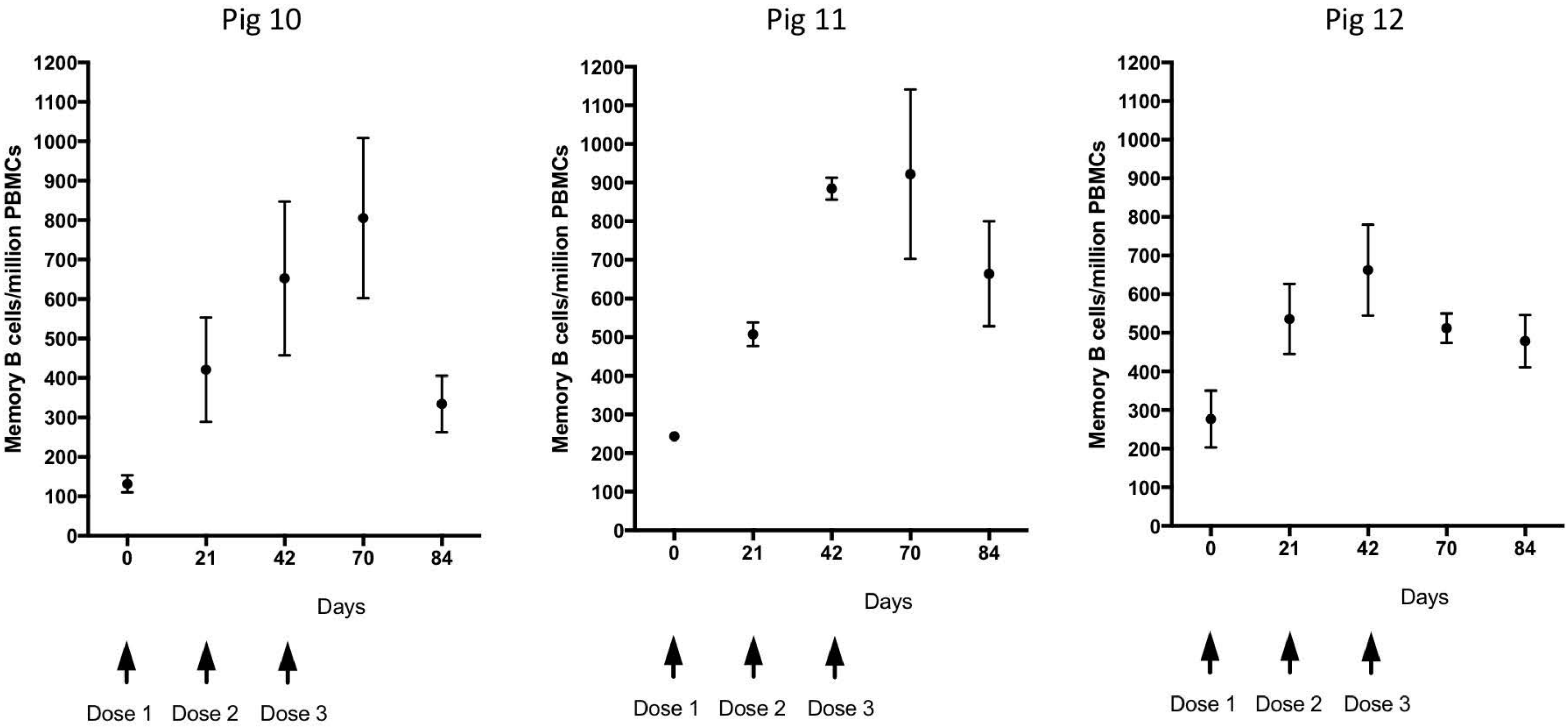

Pig 10
Pig 11
Pig 12
Memory B cells/million PBMCs
0
100
200
300
400
500
600
700
800
900
1000
1100
1200
0
21
42
70
84
Days
Dose 1
Dose 2
Dose 3
